# Study on relationship between elderly sarcopenia and inflammatory cytokine IL-6, anti-inflammatory cytokine IL-10

**DOI:** 10.1186/s12877-018-1007-9

**Published:** 2018-12-12

**Authors:** Yu-Dong Rong, Ai-Lin Bian, Hui-Ying Hu, Yue Ma, Xin-Zi Zhou

**Affiliations:** 1 Health Management Centre, Tianjin First Central Hospital, No. 24 of Fukang Road, Nankai District, Tianjin, 300192 China; 2 Department of Geratology Tianjin First Central Hospital, Tianjin, China; 3 Department of Geratology Yanda International Hospital, Hebei, China

**Keywords:** Elderly, Sarcopenia, Interleukin-6, Interleukin-10

## Abstract

**Background and objectives:**

The pathophysiological mechanism of sarcopenia in the elderly has not yet been fully understood. Here, we aim to explore the relationship between sarcopenia and the inflammatory cytokine interleukin-6 (IL-6), and the anti-inflammatory cytokine interleukin-10 (IL-10) in an elderly population.

**Methods:**

Our study comprised 118 males and 46 females aged between 61 and 90 who had received a general medical examination in Tianjin First Central Hospital. Subjects were divided into a sarcopenia group and a non-sarcopenia group, defined according to the criteria of the Asian Working Group for Sarcopenia (AWGS). We compared body composition, handgrip strength (HS), gait speed (GS), biochemical indexes, levels of IL-6 and IL-10, living habits, and disease status between these groups.

**Results:**

Non-sarcopenia subjects undertook more regular physical exercise than sarcopenia patients. Sarcopenia subjects had higher nutrition risk but lower body mass index (BMI), serum albumin (ALB), triglyceride (TG), and creatinine (Cr) levels compared to non-sarcopenia subjects. Sarcopenia subjects were older and had higher visceral fat tissue (VFA) than non-sarcopenia subjects (*P* < 0.05), along with higher IL-6 and IL-10 levels. Furthermore, IL-6/IL-10 ratios were higher in subjects with sarcopenia (*P* < 0.05). Age, BMI, levels of physical activity, nutritional risk, VFA, IL-6, IL-10, IL-6/IL-10 ratio were independently associated with the presence of sarcopenia in univariate regression analyses. Following adjustment for confounding factors, the presence of sarcopenia was positively correlated with IL-6, IL-10, IL-6/IL-10 ratio and inversely correlated with BMI. Age is associated with increased presence of sarcopenia.

**Conclusions:**

The levels of inflammation cytokine IL-6, anti-inflammatory IL-10 and IL-6/IL-10 ratio were increased in elderly sarcopenia subjects. Sarcopenia was associated with increased levels of inflammatory cytokine IL-6, anti-inflammatory cytokine IL-10, and IL-6/IL-10 ratios.

## Background

The global elderly population is growing as a result of increasing life expectancies. Consequently, chronic age-related morbidities have become a societal burden. Among age-related diseases, sarcopenia is often overlooked. Many studies have reported associations between sarcopenia and adverse outcomes in elderly individuals including increased risk of fall and fracture, impaired ability to perform activities of daily living, disability, increased admission and re-admission rates, and mortality [1]. The consensus on the definition and diagnosis of sarcopenia was published by the European Working Group on Sarcopenia in Older People (EWGSOP) in 2010 and Asian Working Group for Sarcopenia (AWGS) in 2014. In light of this, sarcopenia has garnered increasing interest and attention. Currently, sarcopenia has been accepted as a new geriatric disorder characterised by progressive and generalised loss of skeletal muscle mass and function, along with low muscle strength and poor physical performance**.**

The pathophysiological mechanisms of the onset and progression of sarcopenia are complex with a variety of causes and interactions. But it may include an age-associated state of low-degree systemic inflammation [2, 3] - inflammaging, which is characterised by increasing levels of pro-inflammatory cytokines (including tumor necrosis factor alpha-TNFα and interleukin 6 - IL-6), and decreasing levels of anti-inflammatory cytokines such as interleukin 10 (IL-10) [3].

Studies have reported increased levels of circulating pro-inflammatory cytokines such as IL-6 and TNFα in elderly sarcopenia cases [4, 5]. However, the role of anti-inflammatory cytokines in elderly sarcopenia patients is unknown. IL-10 is currently recognized as an inflammatory and immunosuppressive factor. As an anti-inflammatory cytokine, it suppresses human monocytes and macrophage ability, and the production of pro-inflammatory cytokines including IL-6. To the best of our knowledge, no studies have yet evaluated the potential role of IL-10 in sarcopenia patients. To investigate this question, we measured serum concentrations of the inflammatory cytokine IL-6 and the anti-inflammatory cytokine IL-10, and estimated the association between IL-6 levels, IL-10 levels, and IL-6/IL-10 ratios with sarcopenia in elderly individuals.

## Methods

### Research subjects

A total of 421 elderly subjects (306 male and 115 female) aged between 61 and 90 attended general medical examinations between May 2015 and September 2016 in the Health Management Centre of Tianjin First Central Hospital, China. Eighty-two participants were diagnosed with sarcopenia according to the criteria of AWGS [6]. These subjects included 58 males and 24 females between the ages of 61 and 90 (mean age 76.03 ± 4.33 years).Eighty-two individuals without sarcopenia included 60 males and 22 females between the ages of 61 and 88 (mean age 73.24 ± 5.32 years) were randomly selected from the remaining 339 participants for the non-sarcopenia group.

### Inclusion criteria

All participants were aged over 60 years old and could stand and walk unaided. In the AWGS criteria for sarcopenia in older people [6], diagnosis is based on presentation of criterion 1 (low muscle mass), plus either criterion 2 (low muscle strength) or criterion 3 (poor physical performance) with recommended cutoff values for muscle mass measurements (7.0 kg/m^2^ for men and 5.7 kg/m^2^ for women based on bioimpedance analysis), handgrip strength-HS (< 26 kg for men and < 18 kg for women), and usual gait speed-GS (< 0.8 m/s).

### Exclusion criteria

Exclusion criteria comprised individuals younger than 60 years of age, subjects with tumor, subjects who have been bedridden due to illness such as serious cerebral stroke, heart failure, renal failure, fracture, edema and massive ascites; subjects with severe endocrine diseases that are poorly-managed, subjects who were suffering from infectious diseases, subjects with autoimmune diseases, and subjects with a history of mental illness.

### Medical history and lifestyle

Details on subjects’ medical history and lifestyle were obtained through a questionnaire including smoking and drinking behaviour, physical exercise and medical history. Physical exercise was defined as > 30 min for each activity, > 3 times a week and lasting for more than 6 months. Smoking was defined as people who have smoked for consecutively or cumulatively for six months. Drinking was defined as drinking > 3 times a week over a period of at least 6 months.

### Body measurements

Height, weight and blood pressure (mmHg) were measured by routine. Body mass index (BMI) was calculated using routine measures. Measurement of HS was obtained using a hand-muscle developer (WCS-II, Beijing). Physical performance was determined by usual GS < 0.8 m/s over a course of 4 m. According to the recommendation of AWGS, height-adjusted skeletal muscle mass (ASMI) defined by appendicular skeletal muscle mass (ASM)/height (m)^2^ was used to evaluate the muscle mass. ASM and visceral fat tissue (VFA) were measured by bioelectrical impedance analysis (BIA).

### Nutritional risk

We evaluated the nutritional risk using the mini nutritional assessment short form (MNA-SF). Participants with scores less than or equal to 11 points were considered to be at a risk of malnutrition.

### Laboratory indicators

5 ml of elbow venous blood was drawn in the morning for each subject after at least hours of fasting. Hemoglobin (Hb) and blood biochemistry indicators were tested immediately upon blood-draw. The remaining sera were stored at − 80 °C for later testing of IL-10 and IL-6 levels.

### IL-6 and IL-10 quantification

IL-6 and IL-10 were quantified with competitive inhibition of enzyme-linked immunosorbent assay kits (ELISA; Rapid Bio RB, USA). The sensitivity of detectable concentrations were 0.4 pg/ml for IL-6 and 0.1 pg/ml for IL-10.

### Statistical methods

Continuous variables were expressed by mean ±standard deviation (SD). Categorical variables were described as percentage (%). The differences in means and proportions between different groups were analyzed with t-test and Chi-square tests, respectively. Multiple logistic regression analysis was performed to estimate the odds ratios (OR) and 95% confidence intervals (CIs) for sarcopenia in relation to inflammatory factors. To understand whether potential confounders could affect ORs, we used a multivariate model with adjustment for the following covariates: age, sex, BMI, VFA, hypertension, diabetes, cardiovascular disease, smoking behaviors, drinking behaviour, physical activity, nutritional risk, triglyceride (TG), low-density lipoprotein cholesterol (LDL-C), hemoglobin A1C (HbA1C), Hb, serum albumin (ALB), creatinine (Cr). All statistical analyses were performed using SPSS software version 19.0. A two-tailed *p* value of less than 0.05 was considered to be statistically significant.

## Results

Sample demographics are presented in Table 1. Compared to those without sarcopenia, participants with sarcopenia undertook less regular physical exercise, had lower BMI, ALB, TG and Cr, but higher nutritional risk. Patients with sarcopenia were older on average, and had higher VFA relative to those without sarcopenia.

**Table 1:** Basic characteristics of study sample

Variable	Sarcopenia *n* = 82	Non-Sarcopenia n = 82	*p*
Sex (male/female)	58/24	60/22	0.728
Age	76.03 ± 4.33	73.24 ± 5.32	< 0.001
BMI	23.13 ± 2.78	25.52 ± 2.23	< 0.001
VFA	104.12 ± 18.21	96.01 ± 16.80	0.003
ASMI	6.60 ± 0.75	7.61 ± 0.80	< 0.001
Hypertension (%)	25 (30.49)	22 (26.83)	0.604
Diabetes (%)	12 (14.63)	10 (12.20)	0.647
Cardiac (%)	27 (32.93)	22 (26.83)	0.394
Smoke (%)	13 (15.85)	10 (12.20)	0.500
Drink (%)	12 (14.63)	9 (10.98)	0.483
Sport (%)	9 (10.98)	21 (25.61)	0.015
MNA-SF (%)	32 (39.02)	20 (24.39)	0.044
TG	1.32 ± 0.45	1.56 ± 0.60	0.006
LDL-C	3.14 ± 0.62	3.09 ± 0.61	0.637
HbA1C	5.37 ± 0.95	5.24 ± 1.07	0.433
Hb	124.99 ± 12.05	123.80 ± 17.16	0.618
ALB	41.13 ± 4.55	44.71 ± 4.92	< 0.001
Cr	66.68 ± 14.21	73.16 ± 11.73	0.002

*BMI* body mass index, *VFA* visceral fat tissue, *ASMI* appendicular skeletal muscle index, *MNA-SF* mini-nutritional assessment short-form, *TG* triglyceride, *LDL-C* low-density lipoprotein cholesterol, *HbA1C* hemoglobin A1C, *Hb* hemoglobin, *ALB* serum albumin, *Cr* creatinine

Comparisions of IL-6 and IL-10 levels between sarcopenia and non-sarcopenia patients are summarized in Table 2. Higher levels of IL-6 and IL-10 were observed in participants with sarcopenia (*P* < 0.05). Moreover, higher ratios of IL-6/IL-10 were observed in the sarcopenia group (*P* < 0.05)*.* Univariate analyses were performed investigating the relationship between sarcopenia and IL-6 levels, IL-10 levels and IL-6/IL-10 ratios are presented in Table 3. Independent associations were observed between sarcopenia and age, BMI, physical activity, nutritional risk, VFA, IL-6 levels, IL-10 levels and IL-6/IL-10 ratios. Finally, we performed multivariate analyses to assess the relationship between sarcopenia and IL-6 levels, IL-10 levels and IL-6/IL-10 ratios (Table 4). Following adjustment for potential confounders (age, sex, BMI, physical activity, nutritional risk, VFA), positive associations were present between sarcopenia and IL-6 levels, IL-10 levels, and IL-6/IL-10 ratios. Sarcopenia incidence was found to increase with advancing age whereas there was an inverse correlation between the presene of sarcopenia and BMI.

**Table 2:** Comparison of levels of inflammatory cytokines

Variable	Sarcopenia	Non-Sarcopenia	*p*
IL-6 (pg/ml)	43.80 ± 10.13	27.38 ± 9.53	< 0.001
IL-10 (pg/ml)	4.13 ± 1.03	3.75 ± 1.21	0.032
IL-6/IL-10	9.71 ± 1.43	9.09 ± 1.71	0.013

IL-6: interleukin-6, IL-10: interleukin-10

**Table 3:** Univariate regression analysis for relationship between follow indexes and sarcopenia

Variable	OR (CI 95%)	*p*
Age	1.09 (1.01–1.17)	0.015
sex	0.96 (0.81–1.12)	0.534
BMI	0.80 (0.67–0.96)	0.021
VFA	1.12 (1.02–1.23)	0.039
Hypertension (%)	1.25 (0.56–1.65)	0.092
Diabetes (%)	0.98 (0.72–1.12)	0.063
Cardiac (%)	0.97 (0.90–1.05)	0.362
Smoke (%)	0.83 (0.59–1.05)	0.342
Drink (%)	0.94 (0.79–1.08)	0.128
Sport (%)	0.83 (0.65–0.98)	0.041
MNA-SF(n)	1.05 (1.01–1.35)	0.035
TG	0.92 (0.79–1.16)	0.756
LDL-C	0.83 (0.53–1.14)	0.665
HbA1C	0.94 (0.82–1.10)	0.485
Hb	0.77 (0.65–1.03)	0.086
ALB	0.84 (0.73–1.03)	0.053
Cr	0.83 (0.65–1.14)	0.058
IL-10	1.42 (1.25–1.48)	0.005
IL-6	1.20 (1.06–1.40)	0.043
IL-6/IL-10	1.32 (1.12–1.58)	0.023

*OR* odds ratio, *CI* confidence interval, *BMI* body mass index, *VFA* visceral fat tissue, *MNA-SF* mini-nutritional assessment short-form, *TG* triglyceride, *LDL-C* low-density lipoprotein cholesterol, *HbA1C* hemoglobin A1C, *Hb* hemoglobin, *ALB* serum albumin, *Cr* creatinine, IL-6: interleukin-6, IL-10: interleukin-10

**Table 4:** Multivariate regression analysis for relationship between IL-6 levels, IL-10 levels, IL-6/IL-10 ratio and sarcopenia

Variable	OR (CI 95%)	*p*
IL-10	1.14 (1.03–1.29)	0.047
Age	1.11 (1.03–1.17)	0.017
sex	0.87 (0.67–1.14)	0.483
BMI	0.83 (0.69–1.03)	0.032
VFA	0.63 (0.51–1.12)	0.271
IL-6	1.32 (1.07–1.55)	0.021
IL-6/IL-10	1.76 (1.56–1.91)	0.035
MNA-SF	1.13 (0.82–1.45)	0.312
Sport	0.79 (0.64–1.19)	0.376

*OR* odds ratio, *CI* confidence interval, *IL-10* interleukin-10, *BMI* body mass index, *VFA* visceral fat tissue, *IL-6* interleukin-6, *MNA-SF* mini-nutritional assessment short-form

## Discussion

In the current study, we performed a cross-sectional analysis of serum concentrations of the inflammatory cytokine IL-6 and the anti-inflammatory cytokine IL-10, and IL-6/IL-10 ratios in the context of sarcopenia in elderly individuals. We observed positive associations between sarcopenia and levels of IL-6, IL-10 and IL-6/IL-10 ratios, and a negative association between sarcopenia and BMI.

Recent years have seen increased interest in the potential role of inflammatory mediators in sarcopenia. Evidence exists for a relationship between sarcopenia and age-associated inflammation (i.e. inflammaging). Others have observed elevated levels of circulating pro-inflammatory markers such as IL-6 and TNFα in elderly sarcopenia cases. Moreover, elevated levels of these proteins have been associated with reduced muscle mass and strength [4, 5, 7–9]. Increased levels of inflammatory cytokines have also been reported to play a role in the functional decline of muscle in elderly individuals. Our observation of increased IL-6 levels in sarcopenia is supported by several analyses, in which correlations between reduced physical performance and increased levels of IL-6 and C-reactive protein (CRP) levels have been reported [8, 10, 11]. One possible mechanism for this relationship may relate to increased muscle catabolism induced by higher levels of inflammatory markers [12]. In adults suffering from rheumatoid arthritis, increased levels of TNFα production coincides with higher rates of protein catabolism [13]. Others have demonstrated a causal link between inflammatory cytokines and reduced muscle mass both in-vivo (rodent models) and in-vitro. Infusion of TNFα and IL-6 in rats have been shown to result in proteolysis and atrophy of skeletal muscle [14, 15]. Moreover, negative correlations have been reported between the rate of protein synthesis in skeletal muscle, and levels of CRP, IL-6 and TNFα receptor-2 [16]. Taken together, these findings suggest a role for low-grade inflammation in the development of sarcopenia.

There are multiple reasons for the observed levels of inflammatory markers in our study, the main reason being an age-associated low-grade chronic inflammatory state (i.e. inflammaging), characterized by increased levels of pro-inflammatory cytokines including TNFα, IL-6 and CRP.

Additionally, it is increasingly recognized that skeletal muscle itself is an important source of inflammatory mediators, collectively known as ‘myokines’. These locally generated cytokines (e.g. IL-6, IL-1b, TNFα, IL-1ra) are expressed in skeletal muscle in elderly individuals, and may be linked to the inflammaging process [17, 18].

Age-related changes in body composition, primarily more fat depositing in abdomen and muscles might also be involved in the observed increase of pro-inflammatory cytokines. The present cross-sectional study also showed sarcopenia subjects had higher VFA than non-sarcopenia subjects. As a major endocrine organ, visceral adipose tissue is responsible for insulin resistance and a chronic systemic inflammation. Visceral adipose tissue could release a wide range of inflammatory cytokines, including TNFα, CRP and IL-6 [19].

Additionally, evidence exists to suggest age-associated inflammation and sarcopenia may be related to the change of IL-10 during aging [20]. IL-10 is an anti-inflammatory cytokine, mainly produced by macrophages, T-helper 2 cells, B-cells and monocytes. IL-10 is responsible for suppressing the pro-inflammatory response in various tissues, including skeletal muscle [21], by suppressing the activation of macrophages and releasing and activating inflammatory cytokines such as IL-6, TNFα, and IL-1β [22, 23]. Hacham et al. (year) showed that IL-10 levels and signaling in skeletal muscle are reduced in aging mice [24] whereas Alvarez-Rodriguez et al. (2012) studies also showed decreasing serum levels of IL-10 in aging humans [25]. Furthermore, the IL-10 homozygous knockout mouse has been shown to display increased levels of nuclear factor kappa-light-chain-enhancer of activated B cells (NF-КB)-induced inflammatory mediators [26]. Furthermore, levels of IL-6 in the IL-10 homozygous knockout mice are significantly increased at 50 weeks. These mice display decreased skeletal muscle strength, skeletal muscle quality and muscle weakness with aging, rendering them attractive models for sarcopenia [27]. However, serum IL-10 levels are quite variable in elderly individuals. Previous studies have demonstrated a significant age-related increase in serum IL-10 [28]. So far, few studies have examined whether the association exists between the antiinflammatory cytokine IL-10 and sarcopenia in elderly individuals. Dong et al. (year) evaluated the serum levels of inflammatory markers in disabled and non-disabled elderly Chinese individuals, and found patients with a disability displayed higher levels of IL-10 compared with the control group. Moreover, increased IL-10 was associated with poor physical performance [29]. In the current study, we observed significantly increased levels of IL-10 in elderly individuals with sarcopenia. Consistent with Dong et al.’s findings, we showed IL-10 levels increased with age [30]. Age-related changes in body composition in sarcopenia, primarily more fat depositing in the abdomen and muscles, may be associated with serum IL-10 levels. According to our results, we propose that IL-10 suppresses pro-inflammatory cytokine production by feedback [31]. The increased IL-10 levels observed in elderly sarcopenia patients might therefore be compensatory, in an attempt to suppress the chronic low level inflammatory state.

In recent years, the ratio of proinflammatory cytokine IL-6 to anti-inflammatory cytokine IL-10 (IL-6/IL-10 ratio) has been used as a reliable marker for measuring inflammatory status [32, 33]. In the present study, we showed that IL-6/IL-10 ratios were higher for elderly individuals with sarcopenia compared to controls. We speculate that the pro-inflammatory cytokine IL-6 increases with the compensatory increasing of the anti-inflammatory cytokine IL-10 to inhibit the continued pro-inflammatory status, but the compensatory increasing of IL-10 cannot completely counteract the increasing levels of IL-6. Ultimately, the pro-inflammatory status is predominant whereas the anti-inflammatory cytokine IL-10 is relatively insufficient in elderly individuals with sarcopenia. However, more studies are needed to better understand the mechanisms involved in this process.

There is a number of limitations to the current study. Firstly, the make-up of the sample comprised a greater proportion of males than females. There is therefore a risk of selection bias influencing the results and conclusions. Although we included sex as a variable in the univariate and multivariate analyses. The associations demonstrated may be different in a more representative population with an equal proportion of males and females. Secondly, the cross-sectional design limits us from identifying a cause-effect association between sarcopenia and IL-6 levels, IL-10 levels and IL-6/IL-10 ratios. Prospective studies are therefore required. The relatively small sample size of our study poses an additional limitation. To our knowledge, this is the first study to evaluate the relationship between sarcopenia and IL-6 levels, IL-10 levels and IL-6/IL-10 ratios. It is important to note that recombinant IL-10 in humans has been deemed safe in clinical trials for the treatment of autoimmune and neurodegenerative disorders. Thus, IL-10 may be a promising therapeutic candidate for preventing sarcopenia in elderly individuals.

## Conclusion

In conclusion, we propose that elderly individuals with sarcopenia exhibit a compromised “inflammatory status” characterized by higher IL-6, IL-10 concentrations and IL-6/IL-10 ratios. Moreover, IL-10 may prove to be a promising therapeutic agent for the prevention of sarcopenia in the elderly.

## Abbreviations

ALB: Serum albumin
ASM: Appendicular skeletal lean mass
ASMI: Appendicular skeletal muscle index
AWGS: The Asian Working Group for Sarcopenia
BIA: Bioelectrical impedance analysis
BMI: Body mass index
CI: Confidence interval
Cr: Creatinine
CRP: C-reactive protein.
EWGSOP: European Working Group on Sarcopenia in Older People
GS: Gait speed
Hb: Hemoglobin
HbA1C: Hemoglobin A1C
HS: Handgrip strength
IL-10: Interleukin-10
IL-6: interleukin-6
LDL-C: Low-density lipoprotein cholesterol
MNA-SF: Mini-nutritional assessment short-form
NF-КB: Nuclear factor kappa-light-chain-enhancer of activated B cells
OR: Odds ratio
SD: Standard deviation
TG: Triglyceride
TNFα: Tumor necrosis factor alpha
VFA: Visceral fat tissue
